# Robust watermark technique using masking and Hermite transform

**DOI:** 10.1186/s40064-016-3460-2

**Published:** 2016-10-21

**Authors:** Sandra L. Gomez Coronel, Boris Escalante Ramírez, Marco A. Acevedo Mosqueda

**Affiliations:** 1 Departamento de Ingeniería, Instituto Politécnico Nacional, UPIITA, Av. IPN No. 2580. Col. La Laguna Ticomán, 07340 Mexico, D.F. Mexico; 2 Departamento de Procesamiento Digital de Señales, Universidad Nacional Autóma de México, Mexico, D.F. Mexico; 3 Sección de Estudios de Posgrado e Investigación, Area Telecomunicaciones, Instituto Politécnico Nacional, ESIME Zacatenco, Mexico, D.F. Mexico

**Keywords:** Hermite transform, Image normalization, Perceptive mask, Robustness, Spread spectrum, Watermarking

## Abstract

The following paper evaluates a watermark algorithm designed for digital images by using a perceptive mask and a normalization process, thus preventing human eye detection, as well as ensuring its robustness against common processing and geometric attacks. The Hermite transform is employed because it allows a perfect reconstruction of the image, while incorporating human visual system properties; moreover, it is based on the Gaussian functions derivates. The applied watermark represents information of the digital image proprietor. The extraction process is blind, because it does not require the original image. The following techniques were utilized in the evaluation of the algorithm: peak signal-to-noise ratio, the structural similarity index average, the normalized crossed correlation, and bit error rate. Several watermark extraction tests were performed, with against geometric and common processing attacks. It allowed us to identify how many bits in the watermark can be modified for its adequate extraction.

## Background

Internet is a network that facilitates the distribution of digital information (voice, data, audio, video, and images). Since it is accessible to everybody, the information in it is vulnerable to a wide range of manipulations. Thus, it is important to protect digital contents, so as to reliably exchange information through insecure communication channels, as well as avoiding illegal copies or unauthorized alterations. The watermarks (Katzenbeisser and Petitcolas 2000; Arnold and Schmucker 2003), are one of the available solutions to fight the stated problem, since it is information inserted in the digital content in such manner that it remains imperceptible, robust, and difficult to remove or change (Boland and Dautzenberg 1995); however, it must also be susceptible to detection and extraction in order to be verified. Its function is to provide us with information regarding possible alterations in the document; in the worst-case scenario, it should indicate who the author or copyright holder is.

Imperceptibility, robustness, and security requirements are fundamental in designing a watermark technique. The application fields for watermarks are extensive (Cox et al. 2000), and that is why those requirements vary depending on each specific situation. Some of its applications are concerned with copyright protection that enables the identification of the author of multimedia material, as well as content authentication. Hence, watermarks must contain necessary information that will aid determining digital image integrity. In this case, the watermark should be fragile and invisible, since any modification to the watermarked image should alter the mark. Another application area is concerned with controlling copies, thus avoiding the illegal distribution of copyrighted material. Finally, it can be used to verify the radio and/or TV broadcasts by inserting watermarks in commercial advertisements.

For over a decade, different watermarking techniques have been proposed with the purpose of providing both robustness and reliability. Within these, there are two main classifications: those pertaining to the spatial domain, and those that work in the transform domain. The first ones directly modify the marked pixels so as to insert the watermark. These are simple and use low computational complexity methods in comparison to the second type. They constitute mainly unsafe methods, because the image suffers visible alterations, therefore emphasizing the modified pixels, while degrading the original image quality. Plus, they do not possess robustness against geometric transformations, but only to specific filtering or *JPEG* compressing types (Lee and Chen 2000; Van Schyndel et al. 1994; Nikolaidis and Pitas 1996; Kimpan et al. 2004; Voyatzis and Pitas 1996; Chang and Hsiao 2002).

Aiming to avoid these issues, the transform domain techniques were developed. The most frequently used are: discrete cosine transform (*DCT*) (Cox and Kilian 1996; Lin and Chen 2000; Kung et al. 2002; Zhou et al. 2006), the discrete wavelet transform (*DWT*) (Dugad et al. 1998; Dawei and Wenbo 2004; Dehghan and Safavi 2010; Chang et al. 2010), and the contourlet transform (Candés et al. 2005; Jayalakshmi et al. 2006). These hinder the watermark elimination or modification, since it is inserted in specific elements that guarantee more robustness. We can also find techniques that take into account for the concealment of the watermark, the features of the human vision system (*HVS*) (Wolfgang et al. 1999).

The use of this type of techniques has increased because of the good results shown against intentional and unintentional attacks. For instance, Barni et al. (2001) recommends the masking the watermark by taking into consideration the reduced sensibility of the human eye in detecting noise on the edges, the high and low luminance and brightness, as well as the image’s texturized regions. This study reported satisfactory results against *JPEG’s* compressions and cropping attacks. Baazis proposal (2005)—based on the ideas of Barni et al. (2001)—use contourlet transform instead of (*DCT*). Aiming to endure more geometric attacks, we count with techniques that employ the normalized method for the marking of the image (Dong et al. 2005; Baaziz et al. 2008; Cedillo et al. 2008), hence making it invariant to affine transformations. The described method in Dong et al. (2005) disperses a watermark by using the *DCT*, while applying the normalization process, so as to gain robustness against various attacks. The concealment of the watermark is achieved by using a binary mask, which then is normalized according to the normalization parameters of the original image. The published findings show that it presents robustness against different attacks, such as scaling, rotation, shearing (in both *x* and *y*) direction, median filter, and *JPEG* compression. The length of the watermark is 50 bits (pseudo-random sequence). Another work that uses the normalization process described in Cedillo et al. (2008) has very similar ideas as that of Dong et al. (2005); the difference resides in the classification of blocks in the *DCT* domain: it employs texture features in order to obtain the value of the strength control parameter to insert the watermark. Despite the indication that it constitutes a robust method, the result is a *BER* of 0.04 without fighting any attack.

Finally, we can mention newly developed techniques (Tian et al. 2010; Sridevi and Kumar 2011). In Tian et al. (2010) proposal, the Radon transform is used hoping to correct the image’s orientation. Dong et al. (2005) ideas are also applied to disperse the watermark. The great disadvantage of this method rests on the fact that the obtained *PSNR* values are very low (30 dB) for a 50 bits long watermark, even when it shows good results against attacks such as rotation, scale, *JPEG* compression, and median filter ($BER=0$ average for each on four different images). Sridevi et al. (2011) proposed a watermarking method based on normalization, utilizing the *DCT* and the *DWT*, so as to obtain a more robust method, and that can hold an even bigger watermark. The *PSNR* results are too low, which proves that the embedded image quality suffered. The proposal contained in this paper uses the Hermite transform (*HT*), the spread spectrum method to insert the watermark, and a brightness model—feature that distinguishes it from the process described in Dong et al. (2005)—for the masking of the watermark. Its extraction form is a blind method, because it does not require the original image. Also, the possible quantity of bits that can be modified in the watermark extraction is indicated so as to obtain an undisturbed readable image. This paper is divided as follows: “Hermite transform (HT)” section encloses the theory regarding the Hermite transform; “Watermarking algorithm description” section details the proposed algorithm by using a binary mask (values [0, 1]), as well as a perceptive mask that indicates the watermark extraction process; “Test and results” section describes the tests and results obtained after using common processing and geometric attacks against various images: it also evaluates the advantage of applying a perceptive mask and a minimum *BER* value, to indicate that the extracted watermark remains readable. The last section holds the conclusions.

## Hermite transform (HT)

The Hermite transform (Martens 1990a, b) is a special case of polynomial transform, which is a technique of signal decomposition. The original signal *X*(*x*, *y*), where (*x*, *y*) are the coordinates of the pixels, can be located by multiplying the window function *V* (*x* − *p*, *y* − *q*), by the positions *p*, *q* that conform the sampling lattice *S*, Eq.  1:

1 $$\begin{aligned} X(x,y) = \frac{1}{W(x,y)} \sum _{p,q \in S} X(x,y)V(x-p,y-q) \end{aligned}$$

The periodic weighting function is then defined as Eq.  2:

2 $$\begin{aligned} W(x,y)= \sum _{p,q \in S} V(x-p,y-q) \end{aligned}$$

The unique condition that allows the polynomial transform to exist is that the weighting function must be different from zero for all coordinates (*x*, *y*).

The local information within every analysis window will then be expanded in terms of an orthogonal polynomial set. The polynomials $G_{m,n-m} (x,y)$, used to approximate the windowed information are determined by the analysis window function and satisfy the orthogonal condition, Eq. 3:

3 $$\begin{aligned} \int _{-\infty }^{\infty } \int _{-\infty }^{\infty } V^2(x,y)G_{m,n-m}(x,y)G_{j,i-j}(x,y)dxdy = \delta _{ni} \delta _{mj} \end{aligned}$$

for $$n,i=0,1,\ldots , \infty ; m=0, \ldots , n$$ and $$j=0, \ldots , i.$$

The polynomial coefficients $X_{m,n-m} (p,q)$ are calculated by convolving the original image *X*(*x*, *y*) with the filter function $D_{m,n-m} (x,y)=G_{m,n-m}(-x,-y)V^2(-x,-y)$ followed by a sub-sampling in the positions (*p*, *q*) of the sampling lattice *S*: (Eq. 4)

4 $$\begin{aligned} X_{m,n-m}(p,q) = \int _{-\infty }^{\infty } \int _{-\infty }^{\infty } X(x,y) D_{m,n-m} (x-p, y-q) dxdy \end{aligned}$$

The orthogonal polynomials associated with $V^2(x)$ are known as Hermite polynomials: (Eq. 5)

5 $$\begin{aligned} G_{n-m,m}(x,y) = \frac{1}{\sqrt{2^n(n-m)n!}} H_{n-m}\displaystyle {x \atopwithdelims ()\sigma } H_{m}\displaystyle {y \atopwithdelims ()\sigma } \end{aligned}$$

where $H_n(x)$ denotes the Hermite polynomial of order *n*.

In the case of the Hermite transform, it is possible to demonstrate that the filter functions $D_{m,n-m}(x,y)$ correspond to Gaussian derivatives of order *m* in *x* and ${n-m}$ in *y*, in agreement with the Gaussian derivative model of early vision (Young 1985). Moreover, the window function resembles the receptive field profiles of human vision, Eq. 6:

6 $$\begin{aligned} V(x,y)=\frac{1}{2\pi \sigma ^2} \exp \left( \displaystyle -\frac{x^2+y^2}{2\sigma ^2} \right) \end{aligned}$$

Besides constituting a good model for the overlapped receptive fields found in physiological experiments, the choice of a Gaussian window can be justified because they minimize the uncertainty principle in the spatial and frequency domains. The recovery process of the original image consists in interpolating the transform coefficients through the proper syntheses filters. This process is known as inverse polynomial transform, and is defined by Eq. 7:

7 $$\begin{aligned} \hat{X}(x,y)=\sum _{n=0}^{\infty }\sum _{m=0}^{n}\sum _{p,q \in S} X_{m,n-m}(p,q) P_{m,n-m}(x-p, y-q) \end{aligned}$$

The synthesis filters $P_{m,n-m} (x,y)$ of order *m* in *x*, and ${n-m}$ in *y*, are defined by Eq. 8:

8 $$\begin{aligned} P_{m,n-m}(x,y)= \frac{G_{m,n-m}(x,y)V(x,y)}{W(x,y)} \end{aligned}$$

for $$m=0, \ldots , n$$ and $$n=0, \ldots , \infty$$.

In a discrete implementation, the Gaussian window function may be approximated by the binomial window function Eq. 9:

9 $$\begin{aligned} V^{2}(x) = \frac{1}{2^M}\displaystyle {M \atopwithdelims ()x} \end{aligned}$$

with $x=0,\ldots , M$. The orthonormal polynomials discrete associated to binomial window are known as the Krawtchouck’s polynomials Eq. 10:

10 $$\begin{aligned} G_{n}[x]=\frac{1}{\sqrt{\displaystyle {M \atopwithdelims ()n}}} \sum _{k=0}^{n} (-1)^{n-k} \displaystyle {M-x \atopwithdelims ()n-k} \displaystyle {x \atopwithdelims ()k} \end{aligned}$$

with $x,n=0, \ldots , M$. For long values of *M*, the binomial window approaches a Gaussian window, Eq. 11:

11 $$\begin{aligned} \lim _{M \rightarrow 2}\frac{1}{2^M} \left( \displaystyle {M \atopwithdelims ()x+(M/2)} \right) =\frac{1}{\sqrt{\pi }\sqrt{\frac{M}{2}}} \exp \left[ \displaystyle - \left( \frac{x}{\sqrt{\frac{M}{2}}} \right) ^2\right] \end{aligned}$$

Discrete Hermite transform of length *M* approaches to continuous Hermite transform with standard deviation $\sigma =\frac{x}{\sqrt{M/2}}$.

Analysing the case where *M* is even, we have that filter functions and pattern functions can be centered at the origin moving the window $\frac{M}{2}$ points. Thus the filter function are Eq.  12:

12 $$\begin{aligned} D_{n}(x)=G_n \displaystyle \left( {\frac{M}{2} -x }\right) V^2 \displaystyle \left( {\frac{M}{2} -x}\right) \end{aligned}$$

with $x=-(M/2),\ldots , (M/2)$. These functions can be expressed Eq.  13:

13 $$\begin{aligned} D_n \displaystyle \left( {\frac{M}{2} -x }\right) =\frac{(-1)^n}{2^M\sqrt{\displaystyle {M \atopwithdelims ()n}}} \Delta^n \left[ \displaystyle {M \atopwithdelims ()x} \displaystyle {x \atopwithdelims ()n}\right] \end{aligned}$$

Calculating *Z* transform of this filter function, Eq.  14:

14 $$\begin{aligned} d_{n}(Z)= \sum _{x=-M/2}^{M/2} D_{n}(x)z^{-x} \sqrt{\displaystyle {M \atopwithdelims ()n}} \left( {\frac{1-z}{2}}\right) ^{n}\left( {\frac{1+z}{2}}\right) ^{M-n} \end{aligned}$$

with $n=0,\ldots , M$. These filters have advantage that they can be performed applying successively a number of simplest filters $z^{-1}{(1+z)}^2$, $z^{-1}{(1-z)(1+z)}$, $z^{-1}{(1-z)}^2$, with their respective kernels [1 2 1], [−1 0 1] and [1 −2 1].

**Fig. 1:**
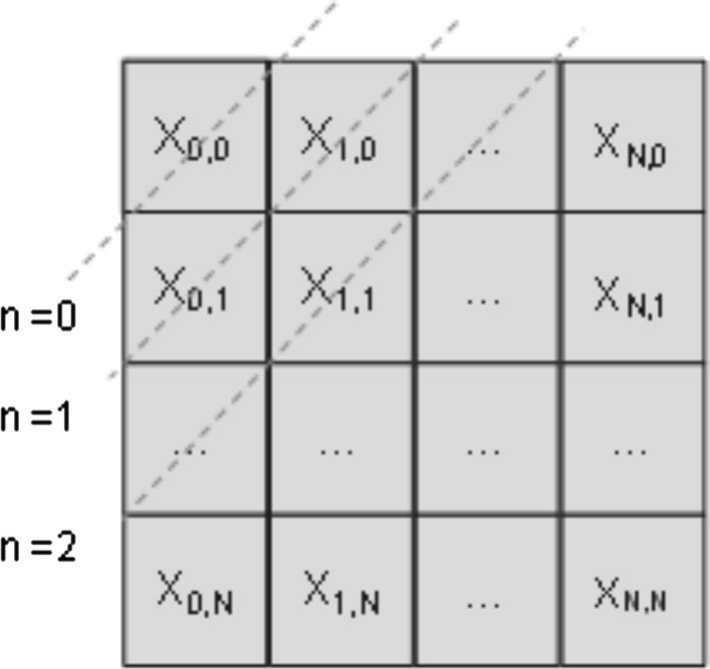
Spatial representation of the Hermite transform coefficients. The *diagonals* represent the coefficients of order zero $n = 0$; the coefficients of order one $n = 1$; and those of order two $n = 2$

Hermite coefficients are arranged as a set of $N \times N$ equal-sized subbands; one coarse subband $X_{0,0}$ representing a Gaussian-weighted image average and detail subbands $X_{n,m}$ corresponding to higher-order Hermite coefficients, as shown in Fig. 1.

## Watermarking algorithm description

The proposed algorithm uses a normalization method based on invariant moments (Hu 1962) in order to prevent alterations in the marked image. It also employs a perceptive mask founded on a brightness model. The watermark is dispersed through a spread spectrum method (*DS-CDMA*; Cox et al. 2000). Each one of these features is described in the following subsections.

### Image normalization

The normalization process of an image *X*(*x*, *y*), with *MxN* dimensions is conformed by:

1. Translation
2. Shearing (both *x* and *y*)
3. Scale

This transformation is performed to gain robustness in the watermark scheme against geometric transformations. The normalization stage happens in the mask, whether it is perceptive or binary, which allows the watermark concealment, thus preventing the visualization of changes in the embedded image.

### Perceptive mask

It is almost impossible to detect a watermark inserted in the frequency domain, whose energy is sufficiently low. However, it is possible to increase the energy of particular frequencies by taking advantage of the human vision system (*HVS*) masking phenomenon. The perceptual masking refers to the fact that information in certain areas of an image is obstructed by more prominent perceptual information in another part of the scene. To improve the robustness of the proposed watermark scheme, we suggest using a perceptive mask during the insertion process instead of that of the original binary mask (Dong et al. 2005). The mentioned perceptive mask is based on the model put forward by Watson (1993). In constructing it, we took into account a luminance-brightness mapping, founded on the model presented by Schouten (1993), who states that the brightness representation remains invariant to the properties of the luminous source, and to the observation conditions. Schouten divides the algorithm for the luminance-brightness mapping in three stages:

1. Multi-scale Representation: This operation is accomplished by the distribution of luminance *L*(*x*). A scaled signal $h_{A}(x,s)$ represents the variations of the reduced luminance with respect to an average level, which is in fact a contrast measure. This operation is carried out in different resolution scales.
  To obtain a scale signal $h_{A}(\vec {x},s)$ from a luminance distribution $L(\vec {x})$ we employ a receptive fields set of different sizes. *s* is the scale and $\vec {x}$ represents position (is a two dimensions vector (*x*, *y*)). Scale signal is a result of interaction between central and peripheral mechanics of receptive field, (Eq.  15)
  15 $$\begin{aligned} h_{A}(\vec {x},s)=f[V_{c}(\vec {x},s),V_{s}(\vec {x},s)] \end{aligned}$$
  And its function is (Eq.  16)
  16 $$\begin{aligned} h_{A}(\vec {x},s)=\alpha \left( {\frac{V_{c}(\vec {x},s)-V_{s}(\vec {x},s)}{V_{c}(\vec {x},s)}}\right) \end{aligned}$$
  where $\alpha$ can be determined by (Eq.  17):
  17 $$\begin{aligned} \alpha =\beta (log(V_{c}(\vec {x},s))-\delta ) \end{aligned}$$
  where $\beta$ and $\delta$ are constants.
2. Scale signals: It consists of transforming the signal $h_{A}(\vec {x},s)$ into an assembled map $A(\vec {x})$ linearly adding up on all spatial scales (Eq.  18):
  18 $$\begin{aligned} A(\vec {x})= \int h_{A}(\vec {x},s)\frac{ds}{s} \end{aligned}$$
  As is necessary finite integral, it would be define low limit *s*− correspond to photo receptors size, and high limit *s*+ would be vision field size. Substituting $s=exp \sigma$, (Eq.  19):
  19 $$\begin{aligned} A(\vec {x})= \int _{\sigma -}^{\sigma +}h_{A}(\vec {x},\sigma )d\sigma \end{aligned}$$
3. Local adjustment of the brightness scale: This adjustment results in the brightness indentation. It can be described as a deflection of the assembled map that leads to a dynamic limited range of the brightness map, which does not seriously affect the local contrast information.

### Discrete algorithm of the luminance-brightness mapping for images

As a pre-processing, the images (*X*(*x*, *y*)) that are going to be employed, must be surrounded by a uniform region with a constant luminance $L_{0}$ , which will be the average value for the image. To avoid unwanted variations, the images are normalized so that the pixels intensity remains in the interval [0, 1]. To carry out the first stage of multi-scale representation, a sampling must take place with distances that increase exponentially, i.e., the (Eq.  19) is a Riemanns sum of terms $h_{A}(x,y,\sigma _{i})$, which are taken in equidistant positions of the scale parameter *s*.

Since the deployed luminance variations only occur in a limited area by a homogeneous region, one can capture those variations by using a limited number of scales. In the discrete expressions, the index *i* indicates the scale and takes the values $i\in {1, 2, 3, \ldots , 9}$. That is why the escalated signal with an index 1, $h_{A}^{(1)} (x,y)$, is the signal with the finest scale, while the one with an index 9, is the signal with the fullest scale. The central and peripheral responses $V_{c}(x,y;s_{i})$ and $V_{s}(x,y;s_{i})$ respectively, are obtained by calculating the convolution between the image and the filters modelling the receptive fields. The ensemble map *A*(*x*, *y*), Eq. 20, is calculated through a sum of the escalated signals $h_{A}^{(i)} (x,y)$ and an offset term $A_{G}$, as expressed in Eq. 21:

20 $$\begin{aligned} A(x,y)=A_{G}+ \ln {2} \sum _{i=1}^{9} h_{A}^{(i)}(x,y) \end{aligned}$$

with

21 $$\begin{aligned} A_{G} \approx \beta (L_{0}-\delta )\tilde{A_{G}} \end{aligned}$$

where $\beta = 0.1$, $\delta = -5.0$ and $\tilde{A_{G}}=1.22$ according to Schouten (1993).

The minimal and maximal values $\hat{A}_{min}(x,y)$ and $\hat{A}_{max}(x,y)$ are calculated through the Eqs. 22 and 23, respectively:

22 $$\begin{aligned} \hat{A}_{min}(x,y) = min \left[ \left[ \ln {2} \sum _{j=0}^{k} h_{A}^{(9-j)}(x,y) \right] +A_{G} \right] , \quad k=0, 1, \ldots , \end{aligned}$$

23 $$\begin{aligned} \hat{A}_{max}(x,y) = max \left[ \left[ \ln {2} \sum _{j=0}^{k} h_{A}^{(9-j)}(x,y) \right] +A_{G} \right] , \quad k=0, 1, \ldots , \end{aligned}$$

Finally, the brightness map is obtained by Eq. 24.

24 $$\begin{aligned} B(x,y)=A(x,y)- \frac{1}{3}-\left( \hat{A}_{min}(x,y)+\hat{A}_{max}(x,y)\right) \end{aligned}$$

### Perceptive mask algorithm

The steps for generating the perceptive mask are:

1. Calculating the coefficients of the image’s Hermite transform *X*(*x*, *y*).
2. Calculating the brightness map *B*(*x*, *y*) of the original image.
3. Calculate the contrast through Eq. 25.
  25 $$\begin{aligned} C=\left[ \sum _{i=1}^{m}\sum _{j=1}^{n-m}C_{i,j}^2\right] ^{\frac{1}{2}} \end{aligned}$$
  where $C_{i,j}$ are the Hermite transform Cartesian coefficients.
4. Calculating the light adaptation threshold, as indicated in Eq. 26.
  26 $$\begin{aligned} C_{thr}= k_{0} \left( C_{min}+ \left| \frac{B^{\alpha }-L_{min}^{\alpha }}{B^{\alpha }+L_{min}^{\alpha }} \right| ^ {\frac{1}{\alpha }} \right) \end{aligned}$$
  where $k_0$ is a constant, $C_{min}$ represents the minimal contrast present when a luminance level $L_{min}$ is present, and when the eye has a maximal sensibility to the contrast (Escalante-Ramirez et al. 2003) and $\alpha$ is a constant that takes values in the interval [0, 1].
5. Generating the perceptive mask *M* (Escalante-Ramirez et al. 2003), according to Eq. 27:
  27 $$\begin{aligned} M= k_{1}max\left( C_{thr}, C^\beta C_{thr}^{1-\beta }\right) \end{aligned}$$
  where:
$$k_1$$ is a constant.

**Fig. 2:**
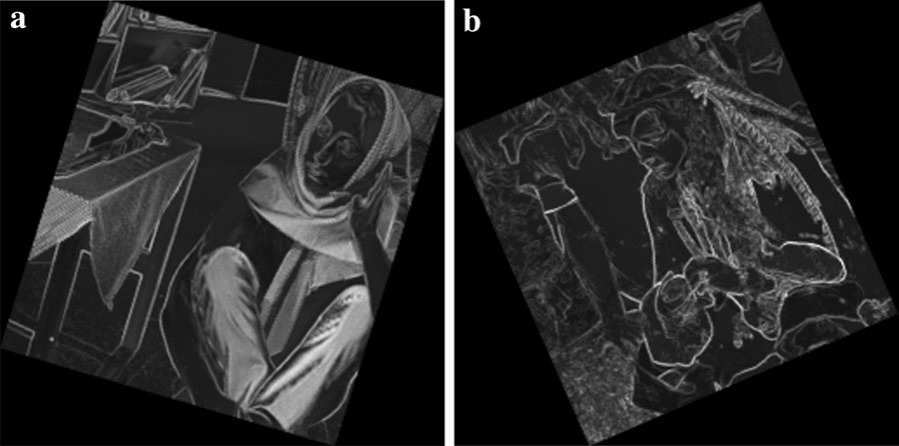
Normalized masks: **a** Barbara (x8), **b** Pirate (x8)

Figure 2 shows the Barbara and Pirate images normalized masks.

### Watermark insertion algorithm

**Fig. 3:**
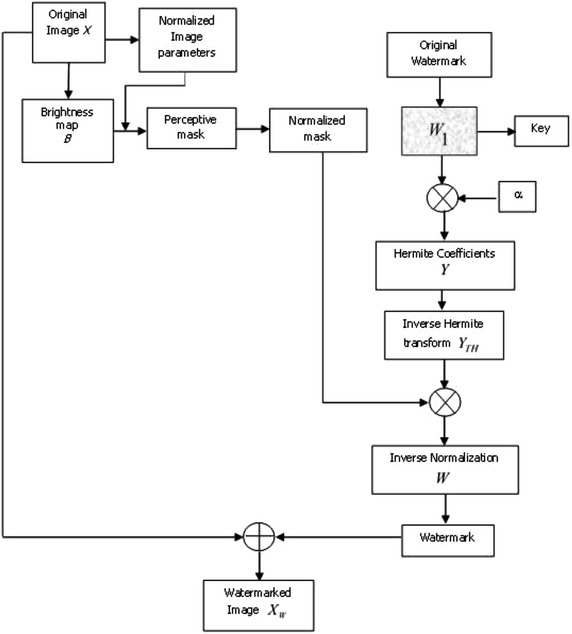
Watermark insertion process scheme

The watermark insertion process is now described, and can be observed in Fig. 3.

1. Normalize the original image *X*(*x*, *y*) to obtain the normalized image $X_{normalized}$.
2. Create the 2*D* watermark, with the same size that the normalized image $X_{normalized}$, according to the following procedure:
  - Generate $p_i$ one-dimensional (1D) binary pseudo-random sequences, by using a private key *k*, where $i=1,\ldots ,l$ and *l* is the number of bits in the message that is used as a watermark, for example we use $l=64$ and $l=104$. Each sequence has values ${-1, 1}$. $p_i$ represents one-dimensional array of arrays. For example if $l=64$, we have $p_1$, $p_2$, $p_3$,…, $p_{64}$. And $p_1$ contains an array $256 \times 256$ size and so on.
  - Create the mark $W_1$ modulating (DS-CDMA) the message with the $p_i$ sequences generated previously, i.e. Eq. 28. In this case the size is $256 \times 256$.
    28 $$\begin{aligned} W_{1}= \sum _{i=1}^{l} (2m_i-1)p_i \end{aligned}$$
    where $m_i$ is the *i*-th bit of the watermark.
  - Generate the null Hermite coefficients $Y_{k,l}$.
  - Create the perceptive mask *M* and normalize it. (If the binary mask is employed, only one template of white pixels must be generated).
  - The insertion of the watermark in the Hermite coefficients $Y_{k,l}$, must be done according to Eq. 29:
    29 $$\begin{aligned} \tilde{Y}_{{k,l}}(i,j)=\alpha W_1 \end{aligned}$$
    where:
$\alpha$ is a strength control parameter to insert the watermark, $W_1$ is the modulated watermark, $\tilde{Y}{_{k,l}}$ is the modified coefficient and (*i*, *j*) are the pixels coordinates.
  - Calculate the inverse transform of the coefficients to obtain $Y_{HT}$.
  - Multiply $Y_{HT}$ with the perceptive mask *M* thus obtaining the final watermark, Eq. 30:
    30 $$\begin{aligned} W_f=Y_{HT}*M \end{aligned}$$
3. Apply the inverse normalization process to $W_f$ so as to obtain *W*.
4. The final watermark is additively inserted in the original image, Eq. 31:
  31 $$\begin{aligned} X_m=X+W \end{aligned}$$

### Watermark extraction algorithm

The watermark extraction method is blind, because it is a correlated one. It consists in:

1. Applying the normalization process in the embedded image $X_m$ to obtain $\tilde{X}_{m}$.
2. Decoding the message of $\tilde{X}_{m}$ as follows:
  - Generate the patterns $p_i$, by using the same key *k* and the same procedure stated in step 2 of the watermark insertion process.
  - Calculate the *HT* of $\tilde{X}_{m}$, to get the coefficient ${Z}{_{k,l}}$.
  - Decode the message (watermark) bit by bit, using a correlated detector between the patterns $p_i$, and the coefficient ${Z}{_{k,l}}$, Eq. 32:
    32 $$\begin{aligned} \ m_i= \left\{ \begin{array}{cl} \displaystyle 1 &{}\quad \text{ corr } \ge 0 \\ 0 &{}\quad \text{ otherwise } \end{array} \right. \end{aligned}$$
    where *corr* represents the correlation between ${Z}{_{k,l}}$ and $p_i$.
  - Convert to its ASCII equivalent, the obtained message from the previous step, and compare it to the original message.

## Test and results

In order to evaluate the performance of the proposal, various tests of insertion, extraction and robustness were performed against common processing and geometric transformations attacks. The perceptive and binary masks (Dong et al. 2005) were used in 26 different images, each one with $512 \times 512$ dimensions. The results employ a watermark length of 64 bits. The metrics employed to evaluate the quality of the embedded images, as well as the watermark extraction, are: peak signal-to-noise ratio (*PSNR*), the structural similarity index (*SSIM*) average, the normalized crossed correlation , and Bit Error Rate (*BER*) was utilized to determine the efficiency of the watermark extraction.

### Watermark insertion and extraction

A watermark (*watermar*) was inserted on each of the 26 images so as to obtain the averages for each metric, as well as the modified bits average. Both the perceptive and the binary mask were applied to determine which one contributes to improving the algorithm performance. The results are shown in Table 1. (The value of $\alpha$ strength control parameter was defined experimentally).

**Table 1:** Averages of the metrics after applying the watermark algorithm to each image. Both binary and perceptive masks were used

Mask	*PSNR* (dB)	*MSSIM*	Correlation	*BER*	Modified bits
Binary	46.3010	0.9927	0.9996	0.0487	3.1923
Perceptive	40.9398	0.9912	0.9988	0.0090	0.6153

**Fig. 4:**
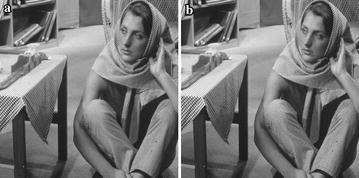
Embedded image *Barbara*, using: **a** binary mask, **b** perceptive mask

**Fig. 5:**
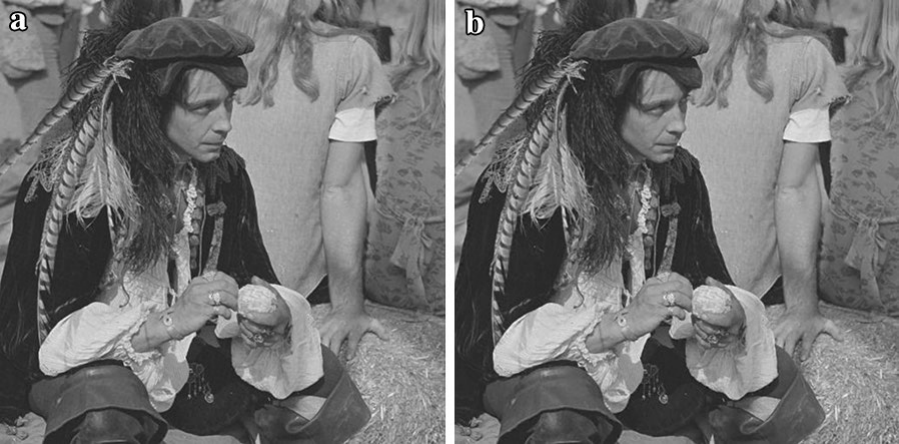
Embedded image *Pirate*, using: **a** binary mask, **b** perceptive mask

After observing Table 1, it is possible to conclude that the binary mask allows for better results in the value *PSNR* (over 45 dB in average) when compared to the value obtained when using the perceptive mask (ca. 40 dB). Regarding the *MSSIM* and the normalized crossed correlation, we got an average of 0.99 in both cases, which indicates that the watermark is not perceptible to the human eye. Nevertheless, concerning the watermark extraction, we obtained better results when using a perceptive mask, because the modified bits average is lower than the unit; while when the binary mask was applied, the modification average was over 3 bits. Also, when analyzing each image results independently, we can observe that the worst extraction case with the binary mask was with a modification of 14 bits, while with the perceptive mask, the maximal modification was with 7 bits. Figures 4 and 5 show only the embedded images using a binary and a perceptive mask, respectively, Barbara and Pirate. However, in Table 2 the metric values for six different images are shown (Lena, Barbara, Pirate, Cambridge2, Cambridge3 and Swan, results are presented in this order), as well as the recovered watermark.

**Table 2:** Metrics results after applying the watermark algorithm to the six images (Lena, Barbara, Pirate, Cambridge2, Cambridge3 and Swan)

Mask	*PSNR* (dB)	*MSSIM*	Correlation	Modified bits	*BER*	Recovered watermark
Binary	46.5676	0.9898	0.9996	4	0.0625	wetå2m‘r
Perceptive	44.9143	0.9925	0.9994	0	0	watermar
Binary	46.2396	0.9919	0.9997	0	0	watermar
Perceptive	40.0937	0.9913	0.9989	0	0	watermar
Binary	46.5337	0.9919	0.9996	0	0	watermar
Perceptive	43.0775	0.9925	0.9992	0	0	watermar
Binary	45.9819	0.9923	0.9996	4	0.0625	waueplas
Perceptive	39.2479	0.9903	0.9982	0	0	watermar
Binary	46.5555	0.9891	0.9996	1	0.0156	wauermar
Perceptive	41.9775	0.9922	0.9989	0	0	watermar
Binary	46.0005	0.9904	0.9996	0	0	watermar
Perceptive	40.4613	0.9924	0.9988	0	0	watermar

The original and the perceptive masks are used

According to the values of Table 2, it is possible to conclude that a better *BER* is obtained by applying the perceptive mask method, given that in these five images, at least, not even one bit of the original mark was modified. There are some images that do exhibit changes, but the performed tests allow us to determine that they can alter up to 2 bits of the recovered mark to ensure that it remains valid. This means that, independently of the mask used, if the watermark extraction shows up to 2 modified bits–the equivalent to a *BER* of 0.03125—it is still readable, and consequently, can be considered a successful extraction.

### Robustness

**Fig. 6:**
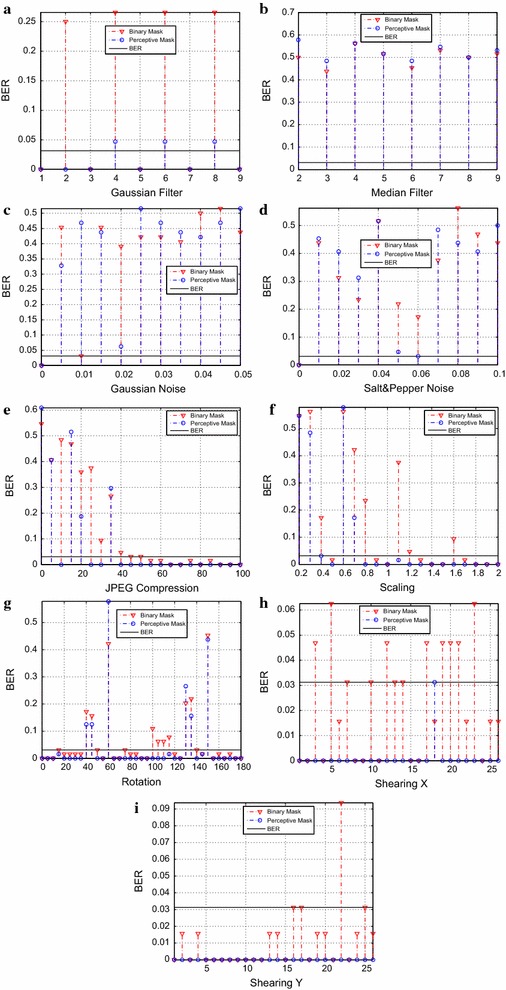
Overcame attacks by the marked image *Barbara*, against: **a** Gaussian filter, **b** median filter, **c** Gaussian noise, **d** salt-and-pepper noise, **e** JPEG compression, **f** scale, **g** rotation, **h** X shearing, **i** Y shearing

**Fig. 7:**
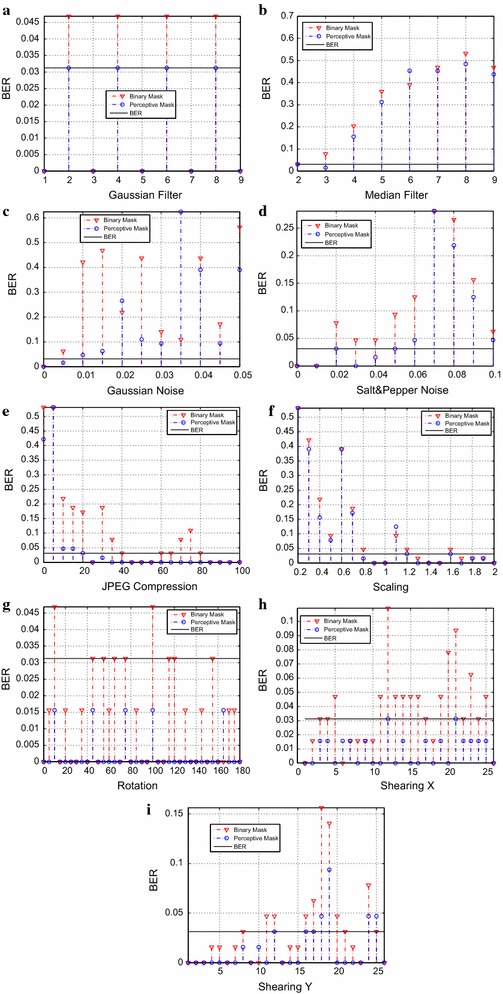
Overcame attacks by the marked image *Pirate*, against: **a** Gaussian filter, **b** median filter, **c** Gaussian noise, **d** salt-and-pepper noise, **e** JPEG compression, **f** scale, **g** rotation, **h** X shearing, **i** Y shearing

To verify the robustness efficacy that the present proposal has, each one of the images had to endure different attacks of common processing and geometric transformations. The attacks executed were: *Gaussian filter*—the window size $N \times N$ went from 1 to 9; *median filter*—the window size $N \times N$ went from 2 to 9; *Addition of Gaussian noise*—the variance went from 0 to 0.005; *Addition of Salt-and-Pepper noise*—in this case, the noise density was modified from 0 to 0.1 with increments of 0.01; *JPEG compression*—the quality factor was applied from 0 to 100 with increments of 5; *Scale*—the used factor of scale went from 20 to 200 % in increments of 10 %; *Rotation*—the variation of the rotation went from 0° to 180°, with increments of 5°. Finally, *shearing*, was applied, whose factor was from 1 to 1 in increments of 0.04. As a sample, Figs. 6 and 7 show the attacks that *Barbara* and *Pirate* images overcame. Both masks were used and the frame of reference is the maximum *BER* that can be obtained in the watermark extraction.

According to the graphics contained in Figs. 6 and 7, we can conclude that using a perceptive mask constitutes a more robust technique against various attacks. To both, the most difficult attack to surmount was the median filter. A *BER* of 0.03125 was taken into account as a limit to consider the watermark extraction successful. Because, even when there is a modification up to 2 bits, it remains readable when converted to its *ASCII* equivalent. In the case of the *Pirate* image, the performance of the algorithm is superior in each attack than that of the binary mask. In the case of the *Barbara* image, we can see that the pattern reoccurs whenever there was a geometric attack.

### Watermark length modification

We performed tests by increasing the length of the watermark up to 104 bits (*GOCS800116MDF*). This was done aiming to prove the bits limit that can be applied for the perceptive mask proposal. Table 3 shows the obtained metrics values for the images *Lena*, *Pirate*, *Blondie* and *Swan*, as well as the recovered mark in each case.

**Table 3:** Metrics results by using a watermark with a 104 bits length, and a perceptive mask

Image	*PSNR* (dB)	*MSSIM*	Correlation	*BER*	Modified bits	Recovered watermark
Lena	42.2444	0.9886	0.9991	0.0192	2	GOCÓ800116MDÆ
Pirate	41.0408	0.9881	0.9988	0.0096	1	GOCS800116MLF
Blondie	40.9906	0.9875	0.9985	0	0	GOCS800116MDF
Swan	38.3915	0.9879	0.9981	0	0	GOCS800116MDF

The results shown in Table 3 demonstrate that the *PSNR* values hold an average of 40 dB, even though that the *Swan* image decreases to 38 dB. Also, the images do not exhibit any visible changes to the human eye. In regards to the watermark extraction, even when not all images got a *BER* of zero—i.e. *Lena* and *Pirate* images—they still can be considered as successful extractions since there was only a modification of 1 bit. Taking into account the same parameters used for the attacks in the previous section, Table 4 reveals the amount of attacks overcame by each one of the embedded images. Every column indicates the type and total amount of attacks applies, as well as the surmounted quantity by each one of the images.

**Table 4:** Watermark algorithm robustness by using a watermark with a length of 104 bits, and a perceptive mask

Image	Gaussian filter (9)	Median filter (8)	Gaussian noise (11)	SP noise (11)	JPEG (21)	Scale (19)	Rotation (37)	X shear. (26)	Y shear. (26)
Lena	5	0	0	0	7	3	24	15	3
Pirate	5	0	0	0	15	6	26	7	13
Blondie	8	0	2	1	14	8	34	21	20
Cisne	9	1	2	3	13	14	36	3	3

Concerning the robustness feature, according to Table 4, the common processing attacks are the ones that are more affected, not so the geometric attacks. The *Swan* image maintains a similar robustness to that obtained in a watermark with a length of 64 bits, except in the shearing. We could state that we possess a robust technique against common processing and geometric transformation attacks, allowing for watermark lengths up to 100 bits, and taking into consideration the impact that the technique exhibited against common processing attacks when the length increased to 104 bits. Other watermarking studies served as a reference to determine the effectiveness of the proposed method. For example, the method described in Cedillo et al. (2008) also uses the normalized approach. It reports a *BER* of 0.04 without facing any attack, and when applying a watermark 64 bits long. In comparison with the present results, in the case of the *Lena* image, the *BER* is 0 with a watermark of the same length; when increased to 104 bits, the *BER* we obtained was of 0.019237, confirming that our method allows for the watermark extraction with a lower rate of erroneous bits and a longer watermark. Now, Tian et al. (2010) employs a watermark method through spread spectrum, the Radon transform, and the *DCT*. It actually proves to be robust, when comparing their results to the normalized approach; they have a $BER=0$ against the rotation attack from 6° to 6° (with increments of 6°); in the scale of 0.5 to 2.0 (with increments of 0.1), as well in some *JPEG* compression and median filter. Nevertheless they obtained an average of 31.5 and 30.0 dB *PSNR* when utilizing a watermark of 50 and 100 bits long.

The procedures described in this paper show that we achieved robustness while facing different types of attacks, and that the *PSNR* values remain close to the 40 dB. The work undertaken by Sridevi et al. (2011) includes a logo as a watermark, which entail more length (4096 *bits*). Their purpose is to manage a robust method, so they calculate the normalization of the image that must be embedded, in order to later break it down in *DWT* coefficients; they work with the median frequency coefficients, to which later they add the calculation of the *DCT*, and then get modified with the watermark. Various attacks are applied: Gaussian noise, rotation, scale, histogram equalization, and contrast modification. Their findings show that it is neither a robust nor a safe technique, since the quality of each image vanishes. They also test the different wavelet coefficients, and the *PSNR* values they obtained were very low (around 13 and 33 dB), without even applying any attack. The work developed by Nah et al. (2012) includes a watermarking technique through the image normalization, and the Correlation Peak Position Modulation (*CPPM*). To use the latter improves the process, instead of using the spread spectrum method, because it remains invariant to the affine transformations. Plus, it facilitates enough capacity to hide specific information (watermark), maximizing it. Their results show that, with a 60 bits long watermark (images with dimensions $512 \times 512$), they acquire $BER=0$ values against rotation attacks (10°, 30°, 45°, 60° and 90°), Salt-and-Pepper noise, and Gaussian noise. However, regarding the visual quality of the watermarked image, the *PSNR* values are lower than 40 dB. For instance, the *Lena* image, *PSNR* = 38.28 dB; the *Baboon* image, *PSNR* = 32.35 dB; and for the *Pepper* image, *PSNR* = 38.76 dB. In our case, every image, with the exception of the image named *Cambridge2*, when a 64 bits watermark was applied, the *PSNR* values were over 40 dB, and as mentioned, exhibited a good performance in regards to robustness. Furthermore, the algorithm described in Singh and Ranade (2013) presents a high capability watermarking technique through the Fast Radial Harmonic Fourier moments (*RHFMs*). While trying to preserve the visual quality of the embedded image, they make use of an adaptive insertion method. That proposal is based on the *RHFMs* calculation in order to improve the invariance properties of the best-preserved moments during the watermark insertion procedures. Thus accomplishing greater robustness against attacks (geometric and common processing). However, the disadvantage they face is that it becomes vulnerable to falsification attacks. The employed images have $256 \times 256$ dimensions, and the resulting *PSNR* values oscillate between 55 and 40 dB for different lengths of the watermark. The robustness is measured with the *BER*. For example, for a 128 bits long mark, in the case of rotation with angles of 5°, 10°, 15° and 20°, the $BER=0$. In our case, even when we use base images with larger dimensions, we acquire $BER=0$ for more rotations. The same happens when facing *JPEG* compression attacks, because in Singh and Ranade (2013), only when the compression factors go up to 30, the *BER* = 0; from there downwards, the *BER* starts to raise. In our approach, the watermark is successfully extracted with lower compression factors.

## Conclusions

This paper presents a watermarking technique that combines the Hermite transform, the normalization process to achieve robustness against geometric transformations, and a perceptive mask. Thus demonstrating that we achieved a robust method that improves its performance when compared to the findings related to the employment of the binary mask (Baaziz et al. 2008). We proved that it is possible to use different watermark lengths (approximately 64 bits to 100 bits), and that even when the robustness may be compromised when facing common processing attacks; if the watermark length increases, the robustness can hold against geometric attacks. Moreover, in order to get a message or short code that enables the identification of the digital image owner, it would not be necessary to have a particularly large watermark. Albeit is true that there are applications with a rather large watermark (Lai 2011; Maity and Kundu 2011)—over 1000 bits, they are usually only pseudo-random sequences that are not extracted as such, but solely detected; or like in the case of the work put forward by Sridevi et al. (2011), which uses a logo as a watermark, and did not overcome any attack implemented to evaluate the technique. In our model, the application entails greater complexity just by trying to extract the message employed as the embedded image. The parameter $\alpha$ value (strength control parameter to insert the watermark) is the one that allows for the changes in the image to remain imperceptible to the human eye. In addition, the perceptive mask helps us to detect those zones that are susceptible to change without making them visible. Our findings show that the reported *PSNR* values are within the 40 dB, which indicates that the image has not suffered significant visual alterations. Furthermore, greater robustness was achieved in every attack, both of common processing and geometric, in comparison to when a binary mask was used. It certainly reaches high *PSNR* values, but it does not guarantee success against attacks. We used different images so as to demonstrate that this technique can be applied to every type of image in gray scale, without limiting to those commonly utilized for this kind of applications (*Lena, Baboon, Barbara, Blondie, Peppers, etc.*). As we took various watermarking studies as a reference, we can confidently claim that the described method complies with the watermark robustness and invisibility, unlike Tian et al. (2010). It does report $BER=0$ against rotation, scale, filtering, and noise attacks; however, the *PSNR* values is low (30 dB), with watermark lengths between 50 and 100 bits. Something similar happens with the approach explained in Cedillo et al. (2008), which has a $BER=0.4$ without any attacks, and with a 64 bits long watermark. It is evident that the improvement in the watermarking algorithms is related to robustness and quality of the embedded image. We have, for instance, the algorithm described in Amiri and Jamzad (2014): it studies the degradation that the watermarked images suffer when printed or scanned, using a model that replicates the distortions produced by the printer and the scanner. They use the *DWT*, the *DCT*, and a genetic algorithm. The lengths of the utilized watermarks are 72, 96, and 128 bits. The metrics employed to evaluate robustness are *PSNR*, *SSIM*, and *BER*. Their findings show a robust algorithm, but due to their images complexity classification-based on the Qaud-tree concept, it may happen that some of those images are wrongly categorized. It results in an unsatisfactory performance of the algorithm. A significant aspect that must be taken into account is that, when dealing with relatively small watermarks, the uncertainty of their recognition is high whenever less than 60 % is properly extracted. Nevertheless, according to our findings, it becomes clear that when an alphanumeric code is used—such as personal identification number—this cannot be applied: a modification of more than 2 bits would alter that code, and it could be mistaken for that of someone else. Such consideration can be taken into account in those algorithms whose watermarks are represented by logos. For example, in the type of works that use a logo as the embedded image, and that consider the *HVS* features (Lai 2011; Maity and Kundu 2011), robust algorithms appear. But, in order to use them as a reference, we have to acknowledge that the watermark extraction should not necessarily be with *BER* = 0 (because of the information quantity used as a watermark); the recognition of the extracted logo would suffice. Therefore, we have determined that our proposal is robust in regards to the bits quantities employed as a watermark, due to the fact that the modified bits—with or without facing attacks—in the embedded images are minimal, or better, they lack modifications altogether. It must be taken into account that the length of the watermark can be augmented and still hold high robustness rates.
